# Premium for an Essay on Physiology, or Medical Chemistry

**Published:** 1850-07

**Authors:** 


PREMIUM FOR AN ESSAY ON PHYSIOLOGY, OR MEDICAL CHEMISTRY.

The following resolution, appended to the report of the committee on medical Literature, was adopted by the Association at the meeting at Cincinnati in May last:

*Resolved,* That the sum of ONE HUNDRED DOLLARS, raised by voluntary contribution, be offered by this Association for the best *experimental* essay on a subject connected either with PHYSIOLOGY, or MEDICAL CHEMISTRY, and that a committee of seven be appointed to carry out the objects of this resolution: said committee to receive the competing memoirs until the first day of March, 1851; the authors' names to be concealed from the committee; and the name of the successful competitor alone to be announced after the publication of the decision.

Dr. FRANCIS G. SMITH, Philad'a, Chairman.
" ALFRED STILLE, Philadelphia.
" FRANKLIN BACHE, "
" L. P. YANDELL, Louisville, Ky.
" JAMES MOULTRIE, Charleston, S. C.
" ROBERT BRIDGES, Phildelphia.
" WASHINGTON L. ATLEE, Philadelphia.

In accordance with the above resolution, the chairman gives notice that the sum of *one hundred dollars* is secured, and will be paid over to the successful competitor, or, if preferred, a gold medal of equal value, bearing a suitable inscription.

The competing memoirs must be transmitted to the chairman, free of expense, and should be designated by some appropriate motto; the author's name accompanying it in a sealed packet, designated in like manner. The successful essay will become the property of the Association, and in case no paper of sufficient merit is offered, the time will be extended for another year.

After the decision of the committee, the sealed packet containing the author's name will be opened in the presence of the ASSOCIATION.

Medical journals throughout the country are requested to give publicity to the abovo notice, and to aid in furthering the wishes of the Association in this respect.

FRANCIS G. SMITH, M. D., Philad'a, Chair.

We are much pleased with this resolution, and hope that it may be productive of good. The subjects chosen for the premium are very interesting ones, and should draw forth a good display of talent. It is earnestly to be desired that the writers shall either make experiments of their own, or verify those that have been made by others. Some of the contributors to this Journal may enter into competition for this premium with fair prospects of success.

